# Identification of influential parameters and conditions in heavy metals adsorption onto Cal-LDH-PC using optimization approaches of RSM and Taguchi

**DOI:** 10.1038/s41598-024-64130-4

**Published:** 2024-06-09

**Authors:** Ava Mohrazi, Reza Ghasemi-Fasaei, Amin Mojiri, Sedigheh Safarzadeh

**Affiliations:** 1 Department of Soil Science, School of Agriculture, Shiraz University, Shiraz, Iran; 2 Department of Civil and Environmental Engineering, Hiroshima University, Higashihiroshima, Japan

**Keywords:** Adsorption, Heavy metals, Response surface methodology, Layer double oxide, Pectin, Environmental sciences, Pollution remediation

## Abstract

Adsorption process plays an important role in the remediation of heavy metals (HMs) from wastewater. A laboratory trial was conducted to investigate effective parameters for improving the bio-adsorption removal of HMs. SEM, EDX, BET, and FTIR techniques were applied to characterize the calcined layer double hydroxide (Cal-LDH), pectin (PC), and Cal-LDH-PC composite prepared from *Licorice pomace*. The adsorption of zinc (Zn) cadmium, nickel (Ni) and lead (Pb) onto the most efficient sorbent was investigated using RSM methodology with operational factors such as concentration, reaction time, sorbent dose, and pH. The results related to FTIR showed that Cal-LDH-PC had the highest number of functional groups. Based on the SEM results Cal-LDH had a low surface area (9.36 m^2^ g^-1^) and a small pore size (9.22 nm). After the modification process (Cal-LDH-PC), the values of surface area and pore size increased by 13-fold (120 m^2^ g^-1^) and 1.5-fold (18 nm), respectively. Cal-LDH had high adsorption performance, more cavities, stability, various functional groups, and excessive carbon and oxygen content, which make it efficient and powerful in removing HMs from wastewater. The optimal condition for achieving the removal efficiency (RE%) values of metals was determined to be 80.79 mg L^−1^, 100 min, 0.167 g L^−1^, and 9 for concentration, reaction time, sorbent dose, and pH, respectively. Maximum adsorption capacity and RE (%) were 300 mg g^−1^ and 99% for Zn. According to the results concentration had a major impact on RE% (except for Ni), while for Ni, adsorbent dose had the most significant impact. The present study introduced Cal-LDH-PC prepared from *Licorice pomace* as a capable, useful and economical sorbent for HMs removal from polluted environments. Taguchi's statistical method is distinguished as an economic method with easier interpretation, while the RSM approach is more accurate, and it can also check the interaction of parameters.

## Introduction

Freshwater is one of the vital resources for human life, but it is exposed to various types of pollution^1^. Common human activities like farming, industry, and commerce produce heavy metals as pollutants^2^. These complex and non-biodegradable compounds are toxic and remain in the environment, causing significant environmental pollution and posing irreparable risks to human life^3,4^. Various methods are used to eliminate metal ions from water, including photocatalytic degradation^5^, membrane separation^6^, and adsorption^7^. However, each strategy has its own drawbacks and advantages. Among all, sorption method is a favorable and green process due to its highly capable, economy, cleanliness, and facile synthesis^8,9^. Layered double hydroxides (LDHs) and their calcination form (layered double oxides (LDOs) or Cal-LDH) are efficient anionic adsorbents for heavy metals (HMs) removal. They are two-dimensional transition metal-based materials coordinated by layers of divalent and trivalent metal cations with interlayer anions. Some researchers believe that Cal-LDH is more efficient than LDH due to its good surface area and sorbent capacity^10^. Ref^11^ Found that the sorbent capacity values of magnesium (Mg) manganese (Mn)-LDH and MgMn- LDO are 5.635 and 8.234 mmol g^−1^, respectively, indicating that the active sites on MgMn- LDO were more than on MgMn-LDH. These results show that Cal-LDH could be applied as an excellent adsorbent for pollutant remediation. The disadvantage of using only LDHs/ Cal-LDH s is that they are not stable for long-term continuous use and cannot be regenerated. To enhance the capacity of LDH, it can be reinforced by recalcitrant material, including compounds derived from agricultural waste, such as biochar, activated carbon, etc.^12^. Ref^13^ Believed that LDH-biochar composites showed promising results in heavy metal removal. In a study, it was found that the application of zinc (Zn) aluminum (Al)-LDH/biochar increased the remediation of pollutants by 44% compared to using ZnAl-LDH alone^14^. To prepare an adsorbent with high capacity and to prevent LDH disintegration, Ref^15^ successfully developed a novel adsorbent called LDH-green adsorbent. Pectin (PC) is one of the green adsorbents that can be obtained from some plants. Previous studies have shown that pectin is used to clean up wastewater, and its composites with other adsorbents, such as chitosan, have the capable to enhance the sorption capacity of chitosan and their stability^16^.

The sorption method strongly depends on some parameters including adsorbent dose, initial pH of aqueous solution, pollutant concentration, and reaction time^17^. In common statistical methods, the one-variable-at-a-time method was usually used in many processes to investigate the impact of the operating parameters, but this method has some drawbacks. This statistical method requires a large number of tests, which is time-consuming and expensive. In addition, this method is incapable of accounting for the interaction between the factors. In recent decades, the Response surface methodology (RSM) and Taguchi techniques have been widely applied as efficient and economic optimization approaches to overcome traditional methods. Both methods reduce costs and saves time by reducing testing runs. These techniques are used for modeling, optimizing, designing experiments, and assessing the impact of independent factors and interactions between them^18,19^. But so far, few studies have examined the advantages and disadvantages of these two methods. In fact, it is necessary to apply optimization methodology to find the optimized conditions as well as optimum level of each factor by best optimization technique^18,19^, However, as far as authors know no study has been conducted to evaluate the advantages and disadvantages of these two techniques in the field of metals adsorption in the environment. In previous research indicated that RSM was more successful to predict removal fluoride than Artificial neural network^20^. Also, in another study that focused on chloride removal, it was found that the predictive power of RSM was higher than 90% ^21^.

The aim of this study were to (i) characterize Cal-LDH-PC, PC, and Cal-LDH prepared from *Licorice pomace*; (ii) evaluate the efficient adsorption capacity of the prepared adsorbents on the removal of some HMs including Zn, cadmium (Cd), lead (Pb), and nickel (Ni) from aqueous solutions; and (iii) optimize the HMs removal based on the best sorbent and determine the optimum level of the operating parameters: HMs concentration, initial pH of the aqueous solution, sorbent dose, and reaction or contact time, (IV) compare optimization capability of RSM and Taguchi approaches in the present study.

## Materials and methods

### Material and instrument

All chemical reagents were used from Merck company products. Some equipment such as Tensor II, Germany, TESCAN-Vega 3, Czech Republic, multi-point Brunauer–Emmett–Teller and Shimadzu AA 670 Japan were utilized in present research.

### Plant material

In this research, *Licorice pomace* was used as pectin raw material. It was obtained from Fars Osareh Iranian Industries Factory, located in Fars province.

### Preparation of different Cal-LDH, PC, Cal-LDH-PC

In the first stage, pectin was synthesized from *Licorice pomace* through a microwave-assisted method^22^. Briefly the *Licorice pomace* was washed, air dried and sieved. Then 30 g of its powder was added to 250 mL distilled water, mixed thoroughly, and the pH reach to 1.5 with HCl. The soiled residues were put in a microwave oven for 3 min. afterwards filtered through Whatman filter paper no. 1, centrifuged dried in an oven at 60 C for 24 h. The white product obtained from this step is called pectin.

In second stage, Mg–Al LDH was synthesized using a facile and co-precipitation method. Mg and Al salts were mixed (4:1 M^+2^:M^+3^) and stirred for 30 min. Then, sodium hydroxide was added dropwise until the pH reached 9.0 and kept for 24 h at 65 °C, centrifuged, filtered, and washed to obtain LDH. In the third stage, LDH was calcined in a muffle furnace at 600 °C to obtain Cal-LDH. In the fourth stage, it was mixed with PC to obtain Cal-LDH-PC. After sonicating the solution containing pectin, Cal-LDHs were added to the solution. Finally, the pH of suspension was adjusted to 10–11, stirred, and filtered to obtain Cal-LDH-PC. All three materials were sieved through a 0.45 μm sieve and kept for next steps.

### Characterization

Fourier transform infrared spectroscopy (FT-IR) (Tensor II, Germany) and SEM with an attached energy dispersive x-ray spectrometer (EDX) (TESCAN-Vega 3, Czech Republic) were used to characterize the functional groups, morphology, and elemental compositions of sorbents produced.

To investigate the sorption capacity (Q_e_) (mg g^-1^) and removal efficiency (RE) (%) of the PC, Cal-LDH, and Cal-LDH-PC, 0.1 g L^-1^ of sorbent were added to 40 ml of water containing 80 mg L^-1^ of four HMs (Zn, Pb, Ni, and Cd as nitrate salt) solution in a 100 ml centrifuge tube. The solution was reacted for 180 min, centrifuged, filtered, and the final HMs concentration in the solution was determined using atomic absorption spectrophotometer (AAS) (Shimadzu AA 670G).

The RE (%) and Q_e_ (mg g^-1^) values of 4 studied HMs was calculated by Eqs. (1 and 2).

1 $$\text{RE }=\frac{{C}_{0 }-{C}_{e}}{{C}_{0}}\times 100$$

2 $${Q}_{e =}\frac{{(c}_{0-}{c}_{e})v}{m}$$

where, C_0_ and C_e_ (mg L^-1^), M (g) and V (L) are initial, final concentrations of HMs, sorbent dose and volume of the solution respectively.

### RSM: the box-behnken design

The operation factors including sorbent dose, contact time, initial pH, and concentration of the pollutant solution were optimized using Box-Behnken Design (BBD). In the RSM method, a mathematical quadratic equation shown in Eq. 3 was used to predict the optimal level of factors and the interaction between them.

3 $$\text{Y}={b}_{0}+{b}_{1}A+{b}_{2}B+{b}_{3}C+{b}_{4}D+{b}_{12}AB+{b}_{13}AC+{{b}_{14}AD+b}_{23}BC+{b}_{24}BD+{b}_{34}CD+{b}_{11}{A}^{2}+{b}_{22}{B}^{2}+{b}_{33}{C}^{2}+{b}_{44}{D}^{2}$$

where Y is the predicted response value; A, B, C and D referred concentration HMs, react time, adsorbent dose and pH respectively. AB, AC, BC, AD and BD showed the interaction of independent variables. Also, A^2^, B^2^, C^2^, D^2^, b_1_, b_2_, b_3_, b_4_, b_12_, b_13_, b_14_, b_23_, b_24_, b_34,_ b_11_, b_22_, b_33_ and b_44_ represent independent factor in square form, linear coefficients of the model, interaction coefficients between the factors and second-order coefficients of the model respectively. Also ${b}_{0}$ Was intercept coefficient.

The total number of experiment's run were calculated follows Eq. (4):

4 $${\text{N}} = {\text{2E }}\left( {{\text{E}} - {1}} \right) \, + {\text{ K}}_{0}$$

where K_0_ and E were the number central point and factors, respectively. Selected levels of operation factors have been belonged to previous researches^23^.

### Taguchi method

According to the Taguchi method, 9 runs are necessary to achieve the optimal levels of variables, while in the traditional method, 81 run are required, which is practically time-consuming. Taguchi uses the signal-to-noise ratio (S/N) in measurable amounts of qualitative characteristics according to the aim of the tests. Which is calculated by Eq. (5): In this equation, n is the number of experiments, and y is the response.

5 $${\text{S}}/{\text{N}}\, = \, - {1}0{\text{ log }}\left[ {\frac{1}{n}\sum {\left( \frac{1}{Y} \right)} ^{{2}} } \right]$$

### Statistical analysis

The experimental design and data were analyzed by using the Design-Expert (version 10.0) and Minitab (version 19) for RSM and Taguchi, respectively. The level and range of operation factors and the design of the experiment based RSM and Taguchi were listed in Tables 1, 2 and 3 respectively.

**Table 1:** Summary of the experimental variables and.

Variable	Unit	Range and level		
		− 1	0	1
A: concentration	(mg L^-1^)	20	90	160
B: react time	min	5	180	92.5
C*:* adsorbent dose	(g L^-1^)	0.1	0.55	1
D:pH	–	5	7	9

**Table 2:** BBD design matrix for the independent variables.

Run	A	B	C	D
1	20	5	0.55	7
2	160	5	0.55	7
3	20	180	0.55	7
4	160	180	0.55	7
5	90	92.5	0.1	5
6	90	92.5	1	5
7	90	92.5	0.1	9
8	90	92.5	1	9
9	20	92.5	0.55	5
10	160	92.5	0.55	5
11	20	92.5	0.55	9
12	160	92.5	0.55	9
13	90	5	0.1	7
14	90	180	0.1	7
15	90	5	1	7
16	90	180	1	7
17	20	92.5	0.1	7
18	160	92.5	0.1	7
19	20	92.5	1	7
20	160	92.5	1	7
21	90	5	0.55	5
22	90	180	0.55	5
23	90	5	0.55	9
24	90	180	0.55	9
25	90	92.5	0.55	7
26	90	92.5	0.55	7
27	90	92.5	0.55	7

**Table 3:** Taguchi design matrix for the independent variables.

Run	A	B	C	D
1	Level 1 of A	Level 1 of B	Level 2 of C	Level 2 of D
2	Level 1 of A	Level 2 of B	Level 2 of C	Level 2 of D
3	Level 2 of A	Level 3 of B	Level 3 of C	Level 2 of D
4	Level 2 of A	Level 1 of B	Level 2 of C	Level 3 of D
5	Level 2 of A	Level 2 of B	Level 3 of C	Level 3 of D
6	Level 2 of A	Level 3 of B	Level 1 of C	Level 2 of D
7	Level 3 of A	Level 1 of B	Level 2 of C	Level 2 of D
8	Level 3 of A	Level 2 of B	Level 2 of C	Level 3 of D
9	Level 3 of A	Level 3 of B	Level 2 of C	Level 2 of D

### Regeneration study

To evaluate the recovery power of the desired adsorbent, the recovery test was performed under optimal conditions.

### Ethics approval/plant guideline statement

It is declared that the present study complies with relevant institutional, national, and international guidelines and legislation. Herein, *Licorice pomace* was obtained from Fars Osareh Iranian Industries Factory, where this processing was done in, follows the mentioned national and international legislation.

## Results and discussions

### Characterization of Pc, Cal-LDH and Cal-LDH-PC

#### Adsorption capacity, SEM-EDX, BET, and FT-IR

**Figure 1:**
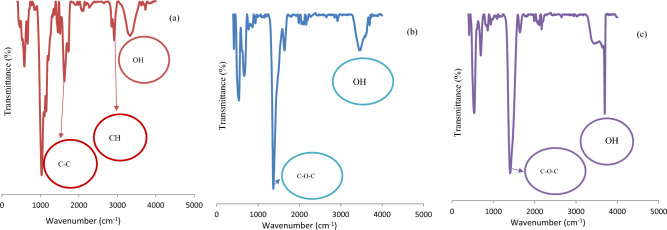
FTIR analysis of cal-LDH-pc (**a**), pc (**b**) and Cal-LDH (**c**).

**Figure 2:**
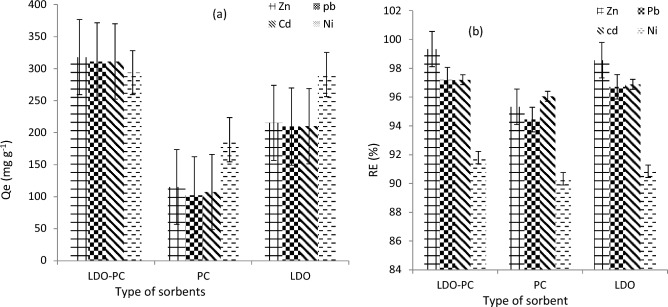
The Qe (mg g^-1^) (**a**) and RE (%) (**b**) Of LDO-PC, PC and LDO.

**Figure 3:**
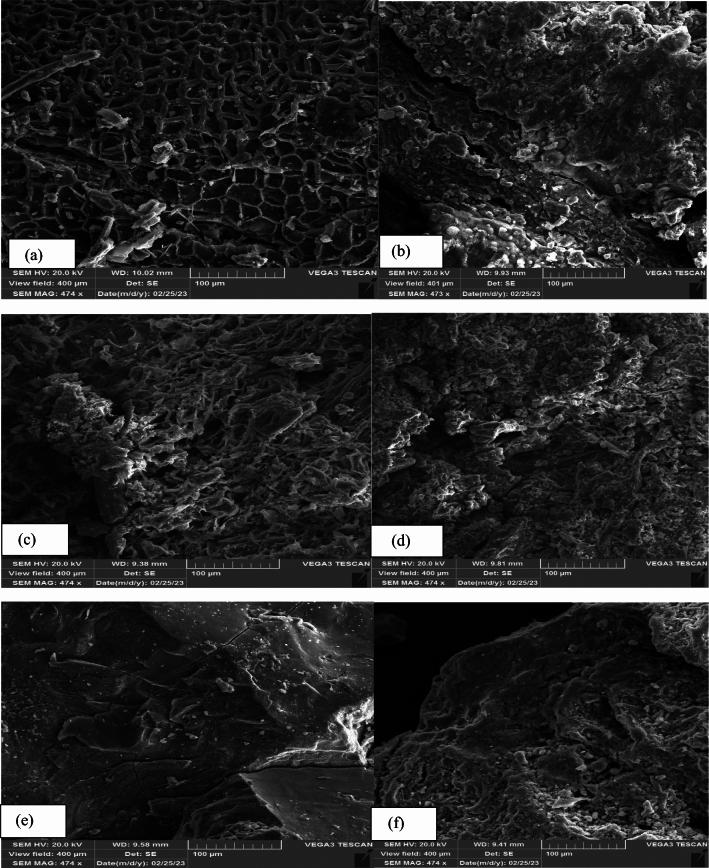
SEM image of LDO-PC before (**a**) and after adsorption (**b**), LDO before (**c**) and after adsorption (**d**) and pectin before (**e**) and after adsorption (**f**).

The textural, morphological and chemical properties of sorbents were characterized and discussed by FT-IR, Qe, and SEM–EDX techniques (Figs. 1, 2, 3 and Table 4). The highest adsorption capacity was observed for Cal-LDH-PC followed by Cal-LDH and then PC. It was found that Cal-LDH -PC had the highest adsorption capacity among the three sorbents, followed by Cal-LDH and then PC. The RE (%) values in present research were 90 to 99%. The maximum RE (%) value referred to Cal-LDH-PC > Cal-LDH > PC for eliminate Zn in waste water also the minimum RE (%) values referred to the PC sorbent. Based SEM analysis Cal-LDH-PC had a porous structure, rough and irregular form, whereas PC and Cal-LDH had smooth surfaces with smaller pore sizes. In conclusion, Cal-LDH-PC had a more cavity compared to the Cal-LDH and PC, providing active sites that facilitate the adsorption of Zn, Cd, Ni, and Pb onto the sorbents^24^. After the sorption process, the shape of the sorbents changed, formation of a white layer, reduction of porosity, and cracks indicated the successful sorption of HMs onto the sorbents^25^. The results of EDX analysis showed Cal-LDH-PC had higher C and O elements as compared to other sorbents. Additionally, the amount of Mg was more than Al, due to the higher cation ratio (MgAl 4:1). After the adsorption process, the amount of HMs on Cal-LDH-PC was more than on Cal-LDH and PC, confirming that Cal-LDH-PC had higher Qe compared to the other sorbent.

**Table 4:** The EDX analysis of Cal-LDH-PC, Cal-LDH and PC sorbent before and after adsorption process.

	Weight% of element before adsorption			Weight% of element after adsorption		
Element	Cal-LDH-PC	Cal-LDH	PC	Cal-LDH-PC	Cal-LDH	PC
C	17.98	13.12	9.92	17.83	11.26	18.12
O	48.73	39.93	38.19	48.82	54.71	49.93
Mg	20.62	20.87	21.31	13.7	28.06	20.87
Al	9.78	9.81	9.04	2.09	4.24	9.81
Ni	0.38	0.12	0.04	3.61	1.08	0.12
Cd	0.67	0.21	0.15	5.85	1.23	0.21
Zn	0.34	0.00	0.17	1.95	0.22	0.10
Pb	0.51	0.95	0.17	6.15	1.20	0.95

FTIR analysis was conducted to evaluate the functional groups of PC, Cal-LDH, and Cal-LDH-PC sorbents. Based on the results, Cal-LDH-PC and PC had the highest and lowest number of functional groups, respectively. The peaks at 3500, 3000, and 1350 cm^-1^ indicated –OH, C-H, and C–O–C, respectively. Also, the peak around the 1600 and 1500 cm^-1^ is related to C=C stretching vibration, C–C and C-N, which illustrate that π–π (pi-pi) interaction between the Cal-LDH-PC, PC and Cal-LDH with HMs that controls the removal of HMs^26^. The band near the 1200 cm^-1^ was appeared in sorbent and after adsorption process shifted to 1020 cm^-1^ the change in position and intensity of bands indicates the interaction between HMs and functional groups of Cal-LDH-PC, PC and Cal-LDH^27^. Additionally, the band below 1000 cm^-1^ was related to the metal -O, which was more abundant in the Cal-LDH -PC sorbent. The peak at 3500–3700 cm^-1^ was related to the residual molecules of water and hydroxyl group, observed in the Cal-LDH sample at this range, while no peak existed in the PC sample. The decreasing intensity of the absorption peak at 1358 cm^−1^ (O–C–O stretching vibration) was observed in Cal-LDH-PC but was not observed in Cal-LDH and PC, this phenomenon related to sorbent synthesis heating temperature. The interlayer carbonate anions of Cal-LDH-PC were decomposed in the 600 °C^28^.

The textural properties of the various sorbents (PC, Cal-LDH, and Cal-LDH-PC) were also analyzed (pore size and surface area are listed in Table 5 also adsorption and desorption curves are shown in Figure S1). The results of BET showed that Cal-LDH had a low surface area (9.36 m^2^ g^-1^) and a small pore size (9.22 nm). After the modification process (Cal-LDH-PC), the values of surface area and pore size increased by 13-fold (116 m^2^ g^-1^) and 1.5-fold (16 nm), respectively. These observations were in agreement with previous results.

**Table 5:** The BET analysis.

	Type of sorbents			
Factor	Cal-LDH-pc	pc	LDH	Unit
Surface area	116	23.11	9.36	(m^2^ g^-1^)
pore diameter	16	11.51	9.22	(nm)

#### Regression model of RSM and Taguchi

The impact of operational factors on the sorption process of Zn, Cd, Ni, and Pb onto Cal-LDH-PC was investigated, based on RSM-BBD (Eqs. 6–9) and Taguchi (Eqs. 10–13). Equations show the resulting model in terms of coded values in relation to RE (%).

6 $${\text{RE}}\% {\text{ of Zn}} = { 97}.{82} + {4}.{\text{52A}} + 0.{\text{64 B}} - 0.{\text{55C}} - {2}.{\text{74AB}} + {1}.{\text{91AC}} - {1}.{\text{44CD}} - {5}.{\text{79A}}^{{2}} - {2}.{\text{58B}}^{{2}}$$

7 $${\text{RE}}\% {\text{ of Cd}} = { 95}.{31} + {3}.{\text{31A}} - {1}.0{\text{4C}} + {1}.{\text{11D}} + {2}.{\text{18AB}} + {11}.{\text{84AC}} + {11}.{\text{24AD}} - {1}0.{\text{29A}}^{{2}} + {1}.{\text{72B}}^{{2}}$$

8 $${\text{RE}}\% {\text{ of Pb}} = { 95}.{79} - {7}.{\text{29A }} - {6}.{\text{24C }} - {21}.{\text{76AB}} + {14}.{\text{76AC}} - {12}.{2}0{\text{AD}} - {9}.{\text{83A}}^{{2}} - {26}.{\text{97B}}^{{2}} - {2}0{\text{C}}^{{2}}$$

9 $$\begin{gathered} {\text{RE}}\% {\text{ of Ni}} = { 98}.{22} + {2}.{\text{52A}} - {4}.{\text{51B}} - {7}.{\text{58C}} + {3}.{6}0{\text{D}} - {9}.{\text{18AB}} + \hfill \\ {25}.0{\text{9AC}} + {14}.{6}0{\text{AD}} - {4}.{\text{88CD}} - {17}.{\text{49A}}^{{2}} - {19}.{\text{29C}}^{{2}} \hfill \\ \end{gathered}$$

10 $${\text{RE}}\% {\text{ of Zn }} = { 7}0.{26 } + {3}.{58} {\text{A }} + {2}.{18} {\text{B }} + 0.{32} {\text{C }} + {4}.{27} {\text{D}}$$

11 $${\text{RE}}\% {\text{ of Cd }} = { 58}.{1 } + {1}.{54} {\text{A }} + {4}.{86} {\text{B }} - 0.{6}0 {\text{C }} + {1}0.{14} {\text{D}}$$

12 $${\text{RE}}\% {\text{ of Pb }} = { 72}.{2 } - {4}.{8} {\text{A }} - {11}.{32} {\text{B }} + {1}.{5} {\text{C }} + {7}.{6} {\text{D}}$$

13 $${\text{RE}}\% {\text{ of Ni }} = { 84}.{8 } + {7}.0{7} {\text{A }} - {9}.{66} {\text{B }} - {9}.{47} {\text{C }} + {8}.{98} {\text{D}}$$

A sign of coefficient indicates how much of an impact it has on the response. (Positive and negative sign represent synergistic and antagonistic impact on response respectively). The BBD was utilized to optimize the experiments, with the response values being the _Qe_ (mg g^-1^) and RE (%) of HMs. The results are shown in Table 6, with the maximum removal efficiency being 99.66%, 99.90%, 98.23%, and 99.59% for Zn, Pb, Cd, and Ni, respectively, which were obtained in runs 7, 24, 17, and 7. The results showed that Zn had the highest RE (%), while Pb had the lowest. Additionally, each parameter had a different impact on the different metals. For example, the lowest RE (%) values were obtained in run 1, and increased with an increase in concentration, reaction time, and sorbent dose, while for Pb, a different response was observed. This result can also be cited for Taguchi (Table 7).

**Table 6:** BBD design matrix for the three independent variables with the actual responses for Q_e_ (mg g^-1^) and RE (%) values.

	RE (%)					Q_e_ (mg g^-1^)		
Run	Zn	Cd	Pb	Ni	Zn	Cd	Pb	Ni
1	80.97	84.81	51.42	72.98	26.10	30.84	28.16	33.901
2	95.675	86.88	76.15	99.2013	281.2	285.43	270.44	288.58
3	88.42	81.89	93.305	78.375	31.26	24.78	33.92	28.5
4	92.16	92.69	31.01	67.8812	281.97	263.07	112.89	197.47
5	95.6889	95.04	79.8667	79.4222	861.2	814.04	718.8	714.8
6	98.43	95.26	79.75	73.21	86.23	82.40	78.18	74.94
7	99.6656	99.8944	96.1889	98.7967	896.99	899.05	865.7	889.17
8	96.66	97.64	68.5	73.08	89.47	89.971	82.843	88.67
9	89.81	94.46	75.59	93.305	34.529	35.756	17.814	33.929
10	96.875	78.24	96.51	67.4125	281.81	261.69	259.23	196.10
11	86.39	71.11	95.435	65.12	28.96	22.62	34.70	12.109
12	96.8919	99.83	67.5625	97.62	281.86	263.52	196.54	198.52
13	95.5111	98.86	58.05	85.84	859.6	808.04	467	707.3
14	95.6056	98.9578	41.6333	78.9	860.45	890.62	374.7	665.2
15	94.11	96.63	38.4444	73.1778	85.911	88.995	34.6	65.86
16	95.1489	99.67	43.18	65.0889	85.634	89.703	19.22	58.58
17	90.14	96.6	98.23	91.15	195.4	197.21	196.46	188.02
18	95.44	80.11	47.201	44.91	1550.87	1444.5	679.8	1109.5
19	84.057	68.44	54.21	24.67	17.409	11.794	11.65	3.42
20	97.0069	99.29	62.2188	78.79	155.211	144.51	99.55	112.55
21	95.7422	96.79	71.08	95.24	156.66	147.65	103.25	132.6
22	95.7178	94.29	65.88	82.0222	156.62	148.09	145.23	134.21
23	93.9	99.04	68.6	99.5567	160.69	162.06	66.92	162.91
24	96.53	99.9011	73	99.5911	159.47	163.47	119.45	162.96
25	96.9911	95.35	97.6856	95.3722	158.71	156.00	159.84	156.06
26	98.35	95.9	91.78	99.6411	163.56	161.48	160.00	163.04
27	98.13	94.68	97.9122	99.6544	159.45	160.41	160.22	163.07

**Table 7:** Taguchi design matrix for the three independent variables with the actual responses for Q_e_ (mg g^-1^) and RE(%) values.

	RE (%)					Q_e_ (mg g^-1^)		
run	Zn	Cd	Pb	Ni	Zn	Cd	Pb	Ni
1	80.97	84.81	51.42	72.98	26.10	30.84	28.16	33.901
2	88.42	81.89	93.305	78.375	31.26	24.78	33.92	28.5
3	95.1489	99.67	43.18	65.0889	85.634	89.703	19.22	58.58
4	93.9	99.04	68.6	99.5567	160.69	162.06	66.92	162.91
5	96.66	97.64	68.5	73.08	89.47	89.971	82.843	88.67
6	95.6056	98.9578	41.6333	78.9	860.45	890.62	374.7	665.2
7	95.675	86.88	76.15	99.2013	281.2	285.43	270.44	288.58
8	96.8919	99.83	67.5625	97.62	281.86	263.52	196.54	198.52
9	92.16	92.69	31.01	67.8812	281.97	263.07	112.89	197.47

The significance of the quadratic equation was justified by considering the interaction effects shown in Tables S2 and S3, and using variance analysis (ANOVA). All suggested RSM models can be used to predict the RE% of HMs via Cal-LDH-PC due to *p*-values less than 0.05, high F-value and non- significant lack of fit (*p*-values > 0.05)^29^. While Taguchi models for Zn and Cd only were capable of predicting the RE% of HMs via Cal-LDH-PC and those for Ni and Pb were not capable of predicting it due to *p*-values more than 0.05. According to Tables 6, 7, each HM had a different response to the operation parameters. based on the RSM results pH had a significant effect on the RE% of Cd and Ni, but not on Zn and Pb. React time had a significant effect only on Zn and Ni, while concentration and sorbent dose had a significant effect on all four studied HMs. The results of Taguchi showed that only concentration had a significant effect on RE% of metals.

### Effects of variable on the RE% of Zn, Pb, Ni and Cd

#### Effect of pH on RE%

pH value is one of the most important factors which have good influence on adsorption process but the efficiency of it depended on some parameters such as type of metal, sorbent and etc. with increasing pH, the RE% value of Ni and Cd significant increased. Increased pH from 5 to 9 increased the RE% value of Ni 85% to 100%. At acidic pH the RE (%) is low which may be due to protonation on the surface of the Cal-LDH-PC in low pH thereby leading to electrostatic repulsion between the sorbent surface and metal^30^.

#### Effect of adsorbent dose on RE%

**Figure 4:**
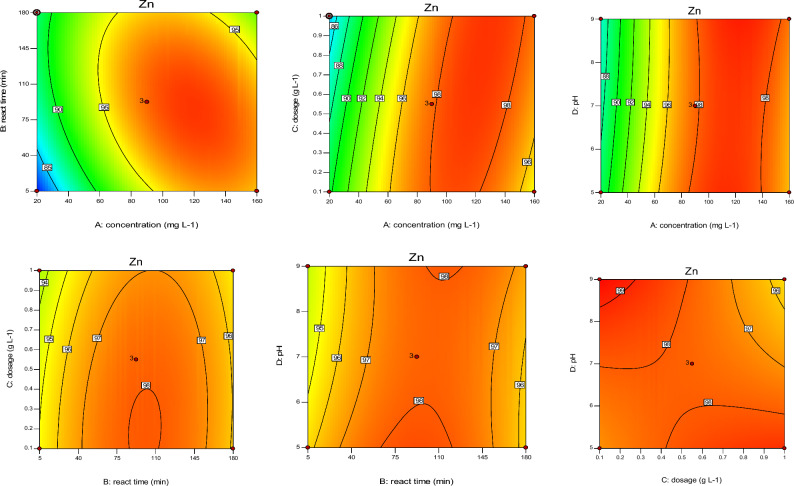
The contour diagram of the interaction of factors impact on RE% of Zn.

**Figure 5:**
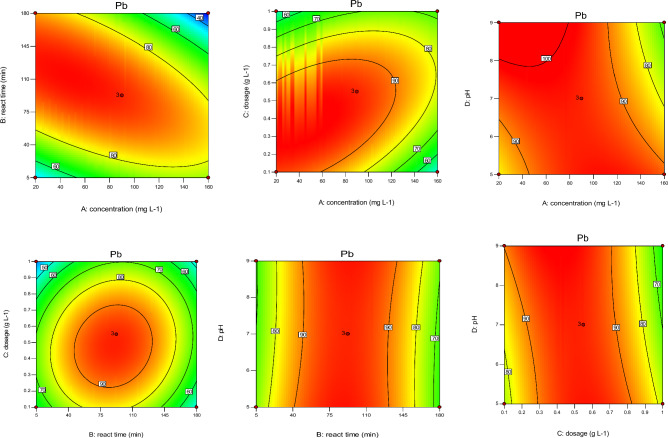
The contour diagram of the interaction of factors impacts on RE% of Pb.

**Figure 6:**
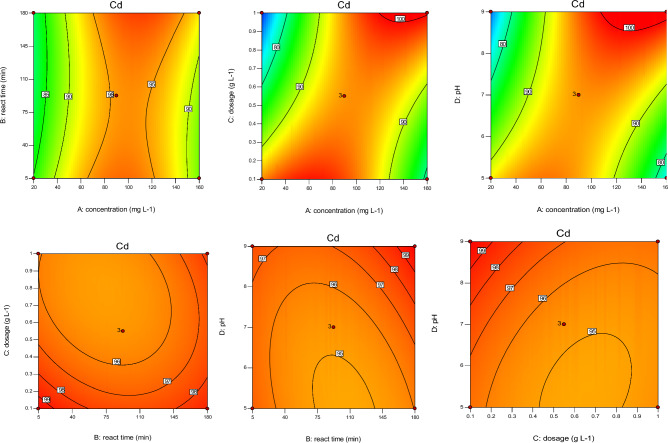
The contour diagram of the interaction of factors impacts on RE% of Cd.

**Figure 7:**
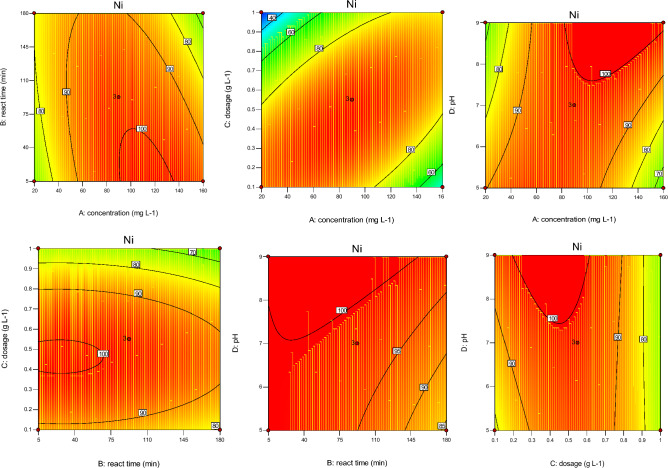
The contour diagram of the interaction of factors impacts on RE% of Ni.

According to Figs. 4, 5, 6, 7, sorbent dosage had significant positive effect on RE (%) value of Cd and Ni. Increased Cal-LDH-PC dosage from 0.2 to 0.7 g L^-1^ increased the RE% value of Ni 80% to 90%. This result is related to the enhance availability of binding sites^31^ However after increased dosage from 0.7 to 1 g L^-1^, decreased the RE% value of Ni 90% to 80%. This result is related to reduction in the amount of HMs contacted per unit mass of Cal-LDH-PC in aqueous solution with the increased sorbent dose. The binding sites could be significantly used at a low dose of Cal-LDH-PC.

#### Effect of initial concentration HMs on RE%

With increasing initial concentration, the RE% value of Ni, Cd and Zn significant increased (except Pb). Also increasing interaction of initial concentration with sorbent dosage had significant increased RE% value of 4 studied metals. While interaction of it with pH had negative effect on RE% value.

#### Effect of contact time on RE%

Based on the Figs. 4, 5, 6, 7 contact time had a positively significant effect on the RE (%) values of Zn (This result is in accordance with Taguchi's results). In other words, an increase reaction time led to an increase in RE% of Zn. But this factor had no significant effect on RE% of Cd and Pb.

#### Effect of interaction independent variable

**Figure 8:**
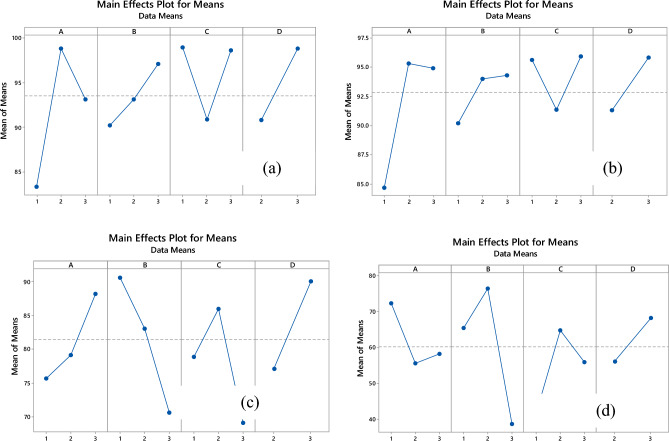
Main effect means for Qe of Zn (**a**), Cd (**b**), Pb (**c**) and Ni (**d**).

The interaction of concentration with reaction time and pH with dose had a negative effect on RE% of Zn, but the interplay of dose with concentration had a positive effect on it. Based on the RSM and Taguchi methods (Figs. 4, 5, 6, 7, 8), the interaction of dose with concentration had a positive impact on it (like Zn, Ni, and Cd). In the Cd test, unlike the other metals, the interaction of operation parameters had a positive effect on RE%. These results indicate that each metal had different behaviors under different conditions, and the RE% of metals strongly depended on the individual and interaction of operation parameters (pH, sorbent dose, concentration, and reaction time). It is clear that concentration (except for Ni) had a noticeable influence on the RE%, while for Ni, the adsorbent dose had the most significant impact on this parameter. Based on the results obtained from Taguchi method, concentration was the most important factor. In other word for Ni and Cd the first rank was concentration based on the Taguchi's results (Fig. 8) (This result is in accordance with the results of RSM). While for pb and Ni, contact time and sorbent dose were the first rank, respectively (Table 8).

**Figure 9:**
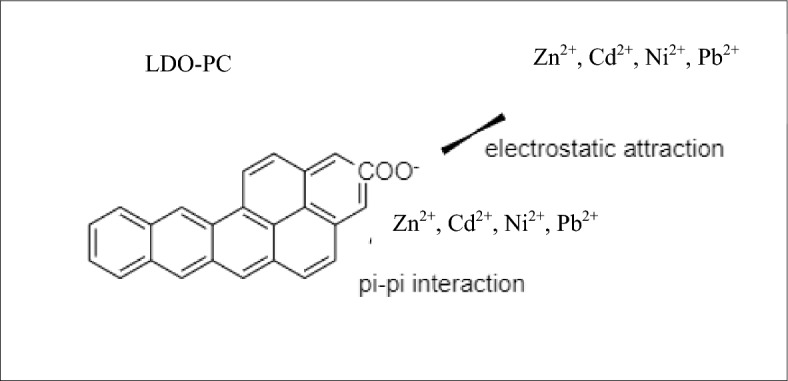
Mechanism of adsorption Zn^2+^, Cd^2+^, Ni^2+^ and Pb^+2^ onto LDO-PC.

**Table 8:** Rank of variable based RSM and Taguchi.

Zn	A	B	C	D
Delta	10.63	4.12	4.57	4.49
Rank-Taguchi	1	4	2	3
Rank-RSM	1	2	3	4
Cd				
Delta	15.48	6.86	8.10	8.02
Rank-Taguchi	1	4	2	3
Rank-RSM	1	2	3	4
Pb				
Delta	16.88	37.85	23.04	12.10
Rank-Taguchi	3	1	2	4
Rank-RSM	1	2	3	4
Ni				
Delta	12.56	19.96	16.85	13.01
Rank-Taguchi	4	1	2	3
Rank-RSM	4	2	1	3

#### RSM optimization

The optimal combination based RSM of the four factors for achieving the RE(%) value of Zn, Pb, Cd, and Ni was found to be 80.79 mg L^-1^, 100 min, 0.167 g L^-1^, and 9 for concentration, react time, sorbent dose, and pH, respectively (as shown in Table 9).

**Table 9:** Verification of adsorption model.

Type of adsorbent	Concentration (mg L^-1^)	React time (min)	Sorbent dose	pH	RE of Zn (%)	RE of Pb (%)	RE of Cd (%)	RE of Ni (%)
Predict-RSM	80.759	100	0.167	9	98.871	97.644	98.585	99.913
Actual-RSM	80.759	100	0.167	9	95.14	96.33	98.6	99.8
Predict-Taguchi	80	100	0.1	9	95.329	98.827	60.151	81.409
Actual-Taguchi	80	100	0.1	9	95.14	96.33	98.6	99.8

Also, the optimal condition based on Taguchi method were 80 mg L^-1^, 100 min, 0.1 g L^-1^ and 9 for concentration, react time, sorbent dose, and pH value, respectively. These results show that both techniques optimize the same conditions; meanwhile RSM optimizes the conditions more accurately. Also it can be concluded that optimal conditions for the operation parameters are required to achieve the highest and the most cost-effective removal of different HMs.

#### Validation of the model

To evaluate the accuracy of the predicted model, experiments were repeated under the optimal conditions, and the results are listed in Table 8. The actual RE% of Zn, Pb, Cd, and Ni were found to be 95.14%, 96.33%, 98.6%, and 99.8%, respectively, while the predicted RE% of HMs were 98.871%, 97.644%, 98.585%, and 99.913% with a small error. While percentage errors obtained from Taguchi were more than 5%. The present research shows that all the results based RSM had a small deviation between experimental and predicted values, and the difference between predicted and experimental values is insignificant. A comparison between the experimental and predicted results based RSM indicated that the error between them is less than 0.3% indicating that the generated model is reliable and powerful more than Taguchi. Therefore, the RSM model can more accurately predict the RE% of the four studied HMs by Cal-LDH-PC in comparison with Taguchi ^32^.

#### Adsorption mechanism

The surface of Cal-LDH-PC, Cal-LDH and PC had potential adsorption active sites for HMs binding. According to FTIR results the -COOH functional groups of sorbents dissociated to COO^−^ that causes chemical bind to Zn, Cd Ni, and Pb via electrostatic force attraction^30^. Also, the tendency of sorbent (Cal-LDH-PC, Cal-LDH and PC) for binding HMs through pi–pi interaction is another mechanism involved in the bio-adsorption of metals^26,33^. Figure 9 is given for better explanation.

**Figure 10:**
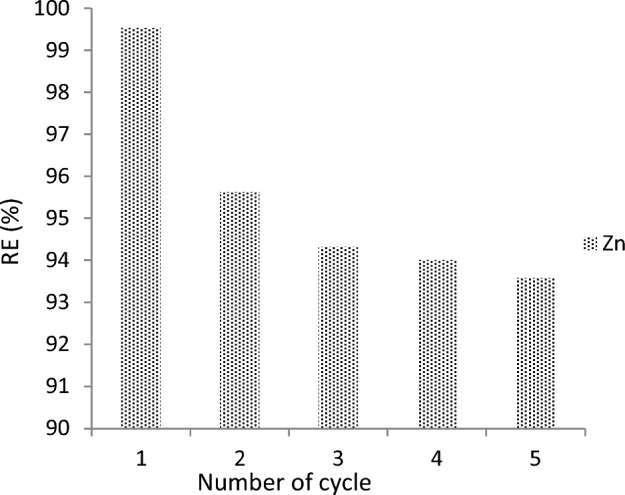
Reusability studies of Cal-LDH-PC. Initial Zn concentration 80 mg L^−1^, contact time, 100 min, and optimal pH value∼9.

#### Regeneration study

One of the important properties that contribute to the reusability of adsorbed HMS is the ability of those materials to be reused. This parameter is determined by^34^ method. The RE (%) of HMs with Cal-LDH-PC decreased in sorption performance after 2 regeneration cycle experiments and after that unchanged (Fig. 10), the RE (%) in the first and final cycle were 99 and 94%, in fact, and the performance remained within an acceptable range. So, the Cal-LDH-PC is relatively stable and has excellent regeneration ability for Zn adsorption.

#### Safe disposal sorbent

Heavy metals are among the important pollutants that have caused global concern. As mentioned in the previous reports, one of the most reliable and safe methods is the use of absorbents, but if these absorbents full of heavy metal are left in nature, it will cause new environmental problems, so we must look for a solution. New to solve this concern, several methods have been reported for this task, which can be mentioned to reuse/regeneration the adsorbents^35^.

## Conclusion

In this study, a low-cost adsorbent called Cal-LDH-PC, prepared from *Licorice pomace*, Al, and Mg, was utilized to efficiently remove Zn, Pb, Cd, and Ni from aqueous solutions. The sorption efficiency of the HMs was investigated and optimized using RSM-BBD and Taguchi. Generally, the Cal-LDH-PC sorbent has a high potential for effective heavy metal removal. The optimal condition of the four operation parameters for achieving the RE% values of Zn, Pb, Cd, and Ni was determined to be 80.79 (mg L^-1^), 100 (min), 0.167 (g L^-1^), and 9 for concentration, reaction time, sorbent dose, and pH, respectively. The optimization results of Taguchi method were similar to RSM method, with the difference that RSM optimized the factors with higher accuracy. The study also showed that the removal efficiency was highly dependent on concentration, reaction time, sorbent dose, and pH, with the effectiveness of each parameter varying depending on the type of HMs and the dose of each factor. Therefore, the preparation of Cal-LDH-PC adsorbent for heavy metal removal is highly recommended due to its inexpensive facile preparation method, and high efficiency. Also, from the comparison of the two statistical methods, it can be seen that the RSM statistical method had higher predictive power and accuracy than the Taguchi. Also, in the RSM statistical method, the interaction of factors can be evaluated, while this is not possible in the Taguchi method.

### Supplementary Information

Supplementary Information.

## Data Availability

The dataset analyzed is available from available from the corresponding author on reasonable request.
