# Supplementary material for: Identification of influential parameters and conditions in heavy metals adsorption onto Cal-LDH-PC using optimization approaches of RSM and Taguchi
Source: Sci Rep. 2024 Jun 9;14:13225. doi: 10.1038/s41598-024-64130-4 (PMC11162457; doi:10.1038/s41598-024-64130-4)
Supplement: Supplementary file 1 — Supplementary Information. [file 41598_2024_64130_MOESM1_ESM.docx]

(a)

Figure S1.  Adsorption and desorption curves.(a),(b) and (c)represented cal-LDH-PC, cal-LDH and PC

Table.S1 ANOVA results of RE% for RSM Model

|  | RE% | | | | | | | | | | | | | |  |  |  |  |  |  |  |  |
| --- | --- | --- | --- | --- | --- | --- | --- | --- | --- | --- | --- | --- | --- | --- | --- | --- | --- | --- | --- | --- | --- | --- |
|  | Sum of |  | Mean | | F | p-value | |  | Sum of | |  | Mean | F | p-value |  |  |  |  |  |  |  |  |
| Source | Squares | df | Square | | Value | Prob > F | |  | Squares | | df | Square | Value | Prob > F |  |  |  |  |  |  |  |  |
| Model _Cd_ | 2076.41 | 14 | 148.32 | | 61.30 | < 0.0001 | | Model _Pb_ | 10511.56 | | 14 | 750.83 | 17.80 | < 0.0001 | significant |  |  |  |  |  |  |  |
| Model _Zn_ | 540.21 | 14 | 38.59 | | 52.09 | < 0.0001 | | Model _Ni_ | 8443.07 | | 14 | 603.08 | 45.73 | < 0.0001 | significant |  |  |  |  |  |  |  |
| A _Cd_ | 131.54 | 1 | 131.54 | | 54.37 | < 0.0001 | | A _Pb_ | 638.57 | | 1 | 638.57 | 15.14 | 0.0021 |  |  |  |  |  |  |  |  |
| A _Zn_ | 245.36 | 1 | 245.36 | | 331.23 | < 0.0001 | | A_Ni_ | 76.08 | | 1 | 76.08 | 5.77 | 0.0334 |  |  |  |  |  |  |  |  |
| B _Cd_ | 1.61 | 1 | 1.61 | | 0.66 | 0.4312 | | B _Pb_ | 20.64 | | 1 | 20.64 | 0.49 | 0.4976 |  |  |  |  |  |  |  |  |
| B _Zn_ | 4.91 | 1 | 4.91 | | 6.62 | 0.0244 | | B_Ni_ | 244.24 | | 1 | 244.24 | 18.52 | 0.0010 |  |  |  |  |  |  |  |  |
| C _Cd_ | 13.09 | 1 | 13.09 | | 5.41 | 0.0384 | | C _Pb_ | 467.09 | | 1 | 467.09 | 11.07 | 0.0060 |  |  |  |  |  |  |  |  |
| C _Zn_ | 3.67 | 1 | 3.67 | | 4.96 | 0.0459 | | C_Ni_ | 690.12 | | 1 | 690.12 | 52.32 | < 0.0001 |  |  |  |  |  |  |  |  |
| D _cd_ | 14.82 | 1 | 14.82 | | 6.13 | 0.0292 | | D _pb_ | 0.031 | | 1 | 0.031 | 7.343E-004 | 0.9788 |  |  |  |  |  |  |  |  |
| D _Zn_ | 0.41 | 1 | 0.41 | | 0.56 | 0.4696 | | D _Ni_ | 155.18 | | 1 | 155.18 | 11.77 | 0.0050 |  |  |  |  |  |  |  |  |
| AB _Cd_ | 19.05 | 1 | 19.05 | | 7.88 | 0.0159 | | AB _Pb_ | 1893.34 | | 1 | 1893.34 | 44.88 | < 0.0001 |  |  |  |  |  |  |  |  |
| AB _Zn_ | 30.06 | 1 | 30.06 | | 40.58 | < 0.0001 | | AB_Ni_ | 337.00 | | 1 | 337.00 | 25.55 | 0.0003 |  |  |  |  |  |  |  |  |
| AC _Cd_ | 560.27 | 1 | 560.27 | | 231.58 | < 0.0001 | | AC _Pb_ | 871.37 | | 1 | 871.37 | 20.66 | 0.0007 |  |  |  |  |  |  |  |  |
| AC _Zn_ | 14.63 | 1 | 14.63 | | 19.75 | 0.0008 | | AC_Ni_ | 2518.03 | | 1 | 2518.03 | 190.92 | < 0.0001 |  |  |  |  |  |  |  |  |
| AD _cd_ | 504.90 | 1 | 504.90 | | 208.69 | < 0.0001 | | AD _pb_ | 595.18 | | 1 | 595.18 | 14.11 | 0.0027 |  |  |  |  |  |  |  |  |
| AD _Zn_ | 2.95 | 1 | 2.95 | | 3.99 | 0.0691 | | AD _Ni_ | 852.42 | | 1 | 852.42 | 64.63 | < 0.0001 |  |  |  |  |  |  |  |  |
| BC _Cd_ | 2.16 | 1 | 2.16 | | 0.89 | 0.3629 | | BC _Pb_ | 111.85 | | 1 | 111.85 | 2.65 | 0.1294 |  |  |  |  |  |  |  |  |
| BC _Zn_ | 0.22 | 1 | 0.22 | | 0.30 | 0.5933 | | BC_Ni_ | 0.33 | | 1 | 0.33 | 0.025 | 0.8769 |  |  |  |  |  |  |  |  |
| BD _cd_ | 2.82 | 1 | 2.82 | | 1.17 | 0.3012 | | BD _pb_ | 23.04 | | 1 | 23.04 | 0.55 | 0.4741 |  |  |  |  |  |  |  |  |
| BD _Zn_ | 1.76 | 1 | 1.76 | | 2.38 | 0.1490 | | BD _Ni_ | 43.91 | | 1 | 43.91 | 3.33 | 0.0931 |  |  |  |  |  |  |  |  |
| CD _cd_ | 1.53 | 1 | 1.53 | | 0.63 | 0.4418 | | CD _pb_ | 190.06 | | 1 | 190.06 | 4.51 | 0.0553 |  |  |  |  |  |  |  |  |
| CD _Zn_ | 8.26 | 1 | 8.26 | | 11.15 | 0.0059 | | CD _Ni_ | 95.11 | | 1 | 95.11 | 7.21 | 0.0198 |  |  |  |  |  |  |  |  |
| A^2^ _Cd_ | 564.87 | 1 | 564.87 | | 233.48 | < 0.0001 | | A^2^ _Pb_ | 515.70 | | 1 | 515.70 | 12.22 | 0.0044 |  |  |  |  |  |  |  |  |
| A^2^ _Zn_ | 178.70 | 1 | 178.70 | | 241.24 | < 0.0001 | | A^2^_Ni_ | 1631.52 | | 1 | 1631.52 | 123.70 | < 0.0001 |  |  |  |  |  |  |  |  |
| B^2^ _Cd_ | 15.70 | 1 | 15.70 | | 6.49 | 0.0256 | | B^2^ _Pb_ | 3879.23 | | 1 | 3879.23 | 91.96 | <0.0001 |  |  |  |  |  |  |  |  |
| B^2^ _Zn_ | 35.54 | 1 | 35.54 | | 47.98 | < 0.0001 | | B^2^_Ni_ | 45.95 | | 1 | 45.95 | 3.48 | 0.0866 |  |  |  |  |  |  |  |  |
| C^2^ _Cd_ | 7.86 | 1 | 7.86 | | 3.25 | 0.0967 | | C^2^ _Pb_ | 2134.29 | | 1 | 2134.29 | 50.59 | <0.0001 |  |  |  |  |  |  |  |  |
| C^2^ _Zn_ | 0.60 | 1 | 0.60 | | 0.81 | 0.3865 | | C^2^_Ni_ | 1984.08 | | 1 | 1984.08 | 150.43 | < 0.0001 |  |  |  |  |  |  |  |  |
| D^2^_Cd_ | 1.93 | 1 | 1.93 | | 0.80 | 0.3891 | | D^2^_pb_ | 9.11 | | 1 | 9.11 | 0.22 | 0.6505 |  |  |  |  |  |  |  |  |
| D^2^_Zn_ | 0.39 | 1 | 0.39 | | 0.52 | 0.4831 | | D^2^_Ni_ | 0.77 | | 1 | 0.77 | 0.058 | 0.8132 |  |  |  |  |  |  |  |  |
| Lack of Fit _Cd_ | 28.29 | 10 | 2.83 | | 7.58 | 0.1221 | | Lack of Fit _Pb_ | 482.05 | | 10 | 48.21 | 3.99 | 0.2171 | not significant |  |  |  |  |  |  |  |
| Lack of Fit _Zn_ | 7.83 | 10 | 0.78 | | 1.47 | 0.4714 | | Lack of Fit_Ni_ | 146.08 | | 10 | 14.61 | 2.40 | 0.3301 | not significant |  |  |  |  |  |  |  |
| Pure Error _Cd_ | 0.75 | 2 | 0.37 | |  |  | | Pure Error _Pb_ | 24.18 | | 2 | 12.09 |  |  |  |  |  |  |  |  |  |  |
| Pure Error _Zn_ | 1.06 | 2 | 0.53 | |  |  | | Pure Error_Ni_ | 12.19 | | 2 | 6.09 |  |  |  |  |  |  |  |  |  |  |
| Cor Total _Cd_ | 2105.44 | 26 |  | |  |  | | Cor Total _Pb_ | 11017.79 | | 26 |  |  |  |  |  |  |  |  |  |  |  |
| Cor Total _Zn_ | 549.10 | 26 |  | |  |  | | Cor Total_Ni_ | 8601.33 | | 26 |  |  |  |  |  |  |  |  |  |  |  |
| R^2^ _Cd_ | 0.98 |  | |  | | |  | R^2^ _Pb_ | 0.95 | |  |  |  |  |  |  |  |  |  |  |  |  |
| R^2^ _Zn_ | 0.98 |  | |  | | |  | R^2^_Ni_ | 0.98 | |  |  |  |  |  |  |  |  |  |  |  |  |
| Adj R^2^ _Cd_ | 0.97 |  | |  | | |  | Adj R^2^ _Pb_ | 0.90 | |  |  |  |  |  |  |  |  |  |  |  |  |
| Adj R _Zn_ | 0.96 |  | |  | | |  | Adj R^2^_Ni_ | 0.96 | |  |  |  |  |  |  |  |  |  |  |  |  |
| Pred R^2^ _Cd_ | 0.92 |  | |  | | |  | Pred R^2^ _Pb_ | 0.70 | |  |  |  |  |  |  |  |  |  |  |  |  |
| Pred R^2^ _Zn_ | 0.91 |  | |  | | |  | Pred R^2^_Ni_ | 0.89 | |  |  |  |  |  |  |  |  |  |  |  |  |
| Adeq Precision _Cd_ | 26 |  | |  | | |  | Adeq Precision _Pb_ | 16.15 | |  |  |  |  |  |  |  |  |  |  |  |  |
| Adeq Precision _Zn_ | 28 |  | |  | | |  | Adeq Precision_rNi_ | 27.91 | |  |  |  |  |  |  |  |  |  |  |  |  |
|  | | | | | | | | | |  | | | | | | |  |  |  |  |  |  |

Table.S2. ANOVA results of RE% for Taguchi Model

|  |
| --- |

Source DF Adj SS Adj MS F-Value P-Value R^2^

Regression_Zn_ 4 147.042 36.7606 2.19 0.008 0.77

Regression_Cd_ 4 289.439 72.360 2.39 0.005 0.70

Regression_Pb_ 4 1253.79 313.448 0.68 0.643 0.40

Regression_Ni_ 4 1247.4 311.85 4.68 0.082 0.82

A_Zn_ 1 54.952 54.9522 3.28 0.008

A_Cd_ 1 10.122 10.122 0.33 0.005

A_Pb_ 1 100.60 100.600 0.22 0.666

A_Ni_ 1 213.8 213.76 3.21 0.148

B_Zn_ 1 23.924 23.9241 1.43 0.298

B_Cd_ 1 119.505 119.505 3.95 0.118

B_Pb_ 1 647.37 647.373 1.40 0.303

B_Ni_ 1 471.1 471.07 7.07 0.056

C_Zn_ 1 0.931 0.931 0.03 0.869

C_Cd_ 1 1.397 1.3974 0.03 0.862

C_Pb_ 1 5.82 5.818 0.01 0.916

C_Ni_ 1 233.6 233.57 3.50 0.134

D_Zn_ 1 27.826 27.8257 1.66 0.267

D_Cd_ 1 156.581 156.581 5.17 0.085

D_Pb_  1 87.08 87.083 0.19 0.687

D_Ni_ 1 122.9 122.94 1.84 0.246

Error_Zn_ 4 67.002 16.7505

Error_Cd_ 4 121.105 30.276

Error_Pb_ 4 1853.73 463.433

Error_Ni_ 4 266.6 66.64

Total_Zn_ 8 214.045

Total_Cd_ 8 410.544

Total_Pb_ 8 3107.53

Total_Ni_ 8 1514.0

|  |
| --- |
